# Combined Transcriptomics, Proteomics and Bioinformatics Identify Drug Targets in Spinal Cord Injury

**DOI:** 10.3390/ijms19051461

**Published:** 2018-05-14

**Authors:** Jure Tica, Elizabeth J. Bradbury, Athanasios Didangelos

**Affiliations:** 1 Imperial College London, Alexander Fleming Building, London SW7 2AZ, UK; j.tica16@imperial.ac.uk; 2 King’s College London, Wolfson CARD, Institute of Psychiatry, Psychology & Neuroscience, London SE1 1UL, UK; elizabeth.bradbury@kcl.ac.uk; 3 Department of Infection, Immunity and Inflammation, University of Leicester, Leicester LE1 7RH, UK

**Keywords:** spinal cord injury, transcriptomics, proteomics, bioinformatics

## Abstract

Spinal cord injury (SCI) causes irreversible tissue damage and severe loss of neurological function. Currently, there are no approved treatments and very few therapeutic targets are under investigation. Here, we combined 4 high-throughput transcriptomics and proteomics datasets, 7 days and 8 weeks following clinically-relevant rat SCI to identify proteins with persistent differential expression post-injury. Out of thousands of differentially regulated entities our combined analysis identified 40 significantly upregulated versus 48 significantly downregulated molecules, which were persistently altered at the mRNA and protein level, 7 days and 8 weeks post-SCI. Bioinformatics analysis was then utilized to identify currently available drugs with activity against the filtered molecules and to isolate proteins with known or unknown function in SCI. Our findings revealed multiple overlooked therapeutic candidates with important bioactivity and established druggability but with unknown expression and function in SCI including the upregulated purine nucleoside phosphorylase (PNP), cathepsins A, H, Z (CTSA, CTSH, CTSZ) and proteasome protease PSMB10, as well as the downregulated ATP citrate lyase (ACLY), malic enzyme (ME1) and sodium-potassium ATPase (ATP1A3), amongst others. This work reveals previously unappreciated therapeutic candidates for SCI and available drugs, thus providing a valuable resource for further studies and potential repurposing of existing therapeutics for SCI.

## 1. Introduction

Severe injury to the mammalian spinal cord causes irreversible tissue damage and in most cases, results in permanent loss of sensorimotor function below the affected site. At the molecular level SCI is characterized by neuronal death and loss of axons, aggressive inflammation, maladaptive tissue remodelling, excessive accumulation of extracellular matrix and scarring [1,2]. These events cause permanent pathological changes at the injury site and prevent neuronal regeneration and axonal growth. With the exception of reducing acute inflammation using corticosteroids, a therapeutic approach which remains controversial [3], followed by chronic rehabilitation physiotherapy, there are no approved therapies for SCI and to date, no drugs can reverse tissue damage or facilitate regrowth of surviving axons through the lesion site. There are few experimental therapies currently under investigation, including antibodies against highly neurotoxic myelin debris proteins (i.e., anti-Nogo antibodies [4]), stabilization of axonal microtubules using paclitaxel/taxol and epothilone B to facilitate the regrowth of damaged axons [5,6] and chondroitinase ABC, an enzyme that digests the growth inhibitory glycosaminoglycan moieties in proteoglycans, which accumulate abundantly in the fibrotic scar that develops after severe SCI and prevent neuronal growth through the injury site [7,8,9]. The complexity of the spinal tissue and the very severe inflammatory, fibrotic and neurodegenerative pathology together with the fact that the SCI patient population is relatively small in comparison to other neurological disorders, make the process of drug discovery difficult.

One possible approach to facilitate the discovery of novel pathological mechanisms and therapeutic targets is to utilize high-throughput -omics such as transcriptomics and proteomics. Contemporary high-throughput methods allow the interrogation of thousands of differentially regulated transcripts or proteins in multiple biological replicates in single quantitative experiments [10]. When combined with rigorous computational analysis of the large data that they return, –omics experiments can provide systems-wide insight into the pathological changes taking place in disease and allow the screening of multi-molecular changes instead of limited focusing towards single genes or proteins.

In this article, we sought to identify previously unappreciated and potentially promising therapeutic candidates for SCI by combining high-throughput transcriptomics and proteomics to profile gene and protein expression changes following clinically-relevant models of rat SCI. To ensure careful filtering of potential therapeutic candidates, we only retrieved molecules that were significantly regulated at the mRNA and protein level in tandem and showed consistent differential expression at 7 days (subacute) and 8 weeks (chronic) post-injury. We subsequently isolated druggable proteins and mined their potential function in SCI. This work could provide the basis for future mechanistic and preclinical studies investigating bioactive molecules with disease-modifying potential in SCI. Importantly, all transcriptomics and proteomics data, as well as the source code for the computational analysis that we developed, are available and freely accessible online via the *Mendeley Data* repository (Elsevier; https://data.mendeley.com) and our analysis is based on highly-cited freely accessible bioinformatics tools.

## 2. Results

### 2.1. Transcriptomics and Proteomics Analysis 7 Days and 8 Weeks after Rat SCI

SCI is a complex disorder which involves multiple different cell types and tissue substrates (neurons and axons, microglia and infiltrating immune cells, astrocytes, vascular cells, meningeal cells and others). It is also affected by the immune privilege of the central nervous system and the vascular limitations of the blood-brain-barrier. Multiparametric high-throughput approaches that examine large-scale transcript and protein changes in tandem can offer a broad understanding of molecular changes in SCI. To this end, we combined high-throughput transcriptomics and proteomics and at two different time-points (7 days and 8 weeks) after SCI to capture consistent and persistent molecular changes post-SCI and to identify proteins with important bioactivity and drug-targeting potential.

First, we performed an intersection of differentially regulated genes identified in a publicly available rat SCI microarray performed recently by Chamankhah and colleagues [11], to identify molecules that were significantly up or downregulated at the mRNA level, both at 7 days and 8 weeks post-SCI. Injury was performed by clip-compression using a 35 g aneurysm clip for 60 s, producing moderate to severe SCI [11]. We chose to use this transcriptomics study because it was performed by an experienced group, it had identical time-points to our proteomics analysis (see below) and importantly, compression SCI shares pathological similarities to contusion injury. This transcriptomics analysis compared uninjured (control) T7 spinal cord segments (*n* = 4) versus injured spinal cord, either at 7 days (*n* = 4) or 8 weeks (*n* = 4) post-injury. All microarray data was made freely accessible online from the authors [11] via the gene expression omnibus (GEO-NCBI: https://www.ncbi.nlm.nih.gov/geo/query/acc.cgi?acc=GSE45006). Moreover, all differentially regulated transcripts from 7 days and 8 weeks post-SCI were downloaded from GEO-NCBI and have been publicly deposited as easily accessible excel files in Mendeley Data: control versus 7 days post-SCI microarray: https://goo.gl/XqbbgN; control versus 8 weeks post-SCI microarray: https://goo.gl/BXYEeT. Only genes that had adjusted *p*-value ≤ 0.05 were accepted in the analysis. 902 were significantly upregulated at both 7 days and 8 weeks versus 835 genes significantly downregulated at both time-points.

Second, to expand the transcriptomics findings to protein expression post-SCI, we used high-throughput proteomics datasets obtained from spinal tissue LC-MS/MS (liquid chromatography-tandem mass spectrometry) performed in our lab. High-throughput analysis at the protein level is biologically important given that transcripts tend to be short-lived in comparison to proteins and mRNA expression does not necessarily reflect protein expression or accumulation at tissue sites, especially given the substantial tissue remodelling that takes place in injured tissues.

To improve the relative enrichment of different protein species and the depth of protein identifications, we used a solubility-based tissue protein subfractionation method previously developed by us, which allows separate analysis of cellular and extracellular proteins by LC-MS/MS and is based on using 0.08% SDS to isolate cellular proteins followed by 4 M guanidine for extracellular matrix and insoluble proteins [10,12,13]. Control (uninjured) versus injured rat spinal cord proteomics comparisons were made again at 7 days and 8 weeks post-SCI, matching the transcriptomics data. Relative estimation of protein abundance in tissue samples by LC-MS/MS was performed using spectral counting [14]. This comparison returned 115 proteins that were significantly upregulated and 149 proteins that were significantly downregulated in tandem, at both 7 days and 8 weeks post-SCI. All protein identification datasets, differentially regulated proteins and statistically analysed spectral counts from 7 days and 8 weeks post-SCI are publicly deposited as easily accessible excel files in Mendeley Data: 7 days proteomics: https://goo.gl/k93LwN; 8 weeks proteomics: https://goo.gl/qYoTJz.

### 2.2. Integration of Transcriptomics and Proteomics Datasets to Identify Persistently Differentially Regulated Molecules

**Figure 1:**
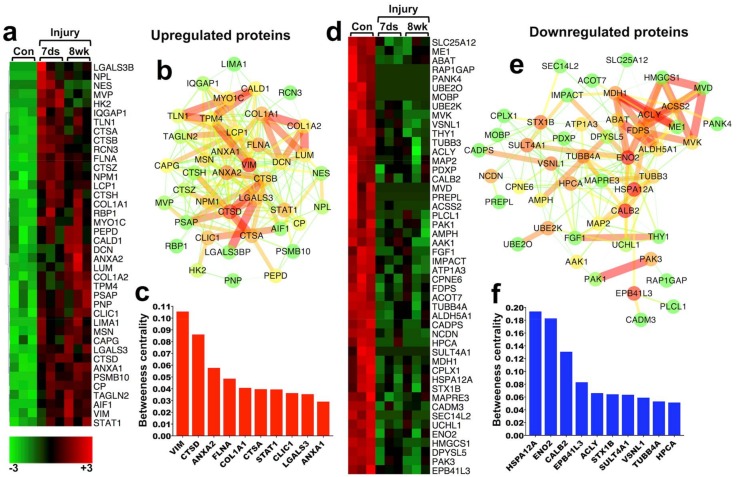
Persistently differentially regulated molecules at the mRNA and protein level 7 days and 8 weeks post-spinal cord injury (SCI): (**a**) 40 molecules that were consistently and significantly upregulated (*t*-test *p* ≤ 0.05, Con vs. 7 ds and Con vs. 8 weeks) in all transcriptomics and proteomics datasets, at both 7 days and 8 weeks post-SCI. Heat-map displays differential protein expression quantified by spectral counting using shotgun proteomics. Heat-map values were normalised from −3 (green; low spectral counts) to +3 (red; high spectral counts). (**b**) The 40 persistently upregulated molecules were collected into a protein-protein interaction network using StringDB and Cytoscape. Node colours indicate protein betweenness centrality (how connected a protein is with others in the network); green nodes: low score; red nodes: high score. The width and colour of edges indicates protein-protein interaction score obtained from StringDB (green and slim: low interaction; red and broad: high interaction). Betweenness centrality and interaction scores were calculated and visualised in Cytoscape. (**c**) Ten upregulated proteins with the highest betweenness centrality score from network (**b**) are depicted. (**d**) 48 molecules that were consistently and significantly downregulated (*t*-test *p* ≤ 0.05, Con vs. 7 ds and Con vs. 8 weeks) in all transcriptomics and proteomics datasets, at both 7 days and 8 weeks post-SCI. Heat-map displays differential protein expression quantified by spectral counting. (**e**) The 48 persistently downregulated molecules were collected into a protein-protein interaction network using StringDB and Cytoscape as above. (**f**) Ten downregulated proteins with the highest betweenness centrality from network (**e**) are depicted.

To filter entities with consistent and persistent differential regulation at the mRNA and protein level and at 7 days and 8 weeks post-SCI, we integrated the transcriptomics and proteomics datasets described above. To ensure stringent selection, we accepted only molecules that were significantly differentially regulated (control vs. injured; *t*-test *p* ≤ 0.05) in all 4 high-throughput datasets (transcriptomics and proteomics, 7 days and 8 weeks post-SCI). This combined analysis returned a filtered signature of only 40 upregulated (Figure 1a–c) and 48 downregulated (Figure 1d–f) molecules, at the transcript and protein level and both at 7 days and 8 weeks post-SCI. These consistent signatures are summarised as heatmaps, which display differential expression from shotgun proteomics (upregulated; Figure 1a and downregulated; Figure 1d) as well as protein-protein interaction networks, which highlight the interconnectivity of the differentially regulated proteins (upregulated; Figure 1b and downregulated; Figure 1e). Figure 1c,f depict 10 upregulated and downregulated proteins respectively, with the highest network betweenness centrality, a measure of how associated and central a protein is in comparison to other network proteins and offers an unbiased assessment of its relative biological importance [15]. Conceivably, proteins with high betweenness centrality and therefore extensive biological association with other proteins, might be considered as good drug targets given that they likely have an important function in the system either in isolation or as part of a functional pathway.

#### 2.2.1. 40 Persistently Upregulated Proteins

The network of the 40 upregulated entities (Figure 1a,b) contains multiple proteins involved in extracellular matrix metabolism, including minor glycoproteins galectin 3 (LGALS3), lumican (LUM) and decorin (DCN) together with annexin A2 (ANXA2) and both alpha chains of collagen-1 (COL1A1 and COL1A2). The upregulated network (Figure 1a,b) also contains cytoskeletal proteins such as LIMA1 (actin-binding), CALD1 (actin-binding caldesmon), AIF1 (microglia/macrophage cytoskeletal protein commonly known as IBA1), LCP1 (plastin-2, T cell actin-binding protein), filamin A (FLNA) and vimentin (VIM) an abundant non-epithelial cytoskeletal protein with key collagen-1 mRNA-stabilising function.

Consistently upregulated proteins also include 5 cathepsins (lysosomal proteases); A (CTSA), B (CTSB), D (CTSD), H (CTSH) and Z (CTSZ) (Figure 1a,b) covering a large spectrum of cellular and extracellular proteolytic substrates. Cathepsin upregulation, proteolytic activity and lysosomal involvement are often associated with inflammatory tissue remodelling and loss of normal tissue function as well as activation of cell death pathways. Cathepsins are typically associated with activated macrophages and other immune cells [16]. The multicatalytic proteasome proteinase PSMB10 and dipeptidase PEPD, the latter with an important function in collagen-1 metabolism, are also present (Figure 1a,b). VIM, CTSD and ANX A2 have high betweenness centrality (Figure 1c), followed by FLNA and COL1A1 (Figure 1c), indicating relative biological importance in the system. The persistent upregulation of these proteins highlights the dominance of inflammation and scarring after SCI, driving pathological matrix remodelling and extensive proteolysis.

#### 2.2.2. 48 Persistently Downregulated Proteins

Unsurprisingly, the network of the 48 persistently downregulated proteins (Figure 1d,e) contains multiple neuronal proteins with direct involvement in synaptic transmission including CPLX1 (complexin-1) and STX1B (syntaxin-1B) involved in synaptic vesicle function, ABAT (mitochondrial aminobutyrate aminotransferase) and ALDH5A1 (mitochondrial aldehyde dehydrogenase), both involved in the degradation of the neurotransmitter GABA, CPNE6 (copine-6; dendrite formation), HPCA (neuron-specific calcium-binding hippocalcin; regulates calcium channels), ME1 (malic enzyme) and AMPH (amphiphysin; involved in synaptic exocytosis). The persistent downregulation of synaptic-associated proteins is likely the result of neurodegeneration following SCI.

The downregulated network also contains 7 proteins involved in cholesterol and lipid synthesis and metabolism including MVD and MVK (mevalonate decarboxylase and kinase respectively), FDPS (farnesyl diphosphate/pyrophosphate kinase), HMGCS1 (catalyses synthesis of mevalonate from acetyl-CoA), ACSS2 (acetyl-CoA synthetase), ACLY (ATP citrate lyase) involved in acetyl-CoA metabolism) and SEC14L2 (supernatant protein factor). The central nervous system-specific heat-shock protein 70, 12A (HSPA12A) has the highest betweenness centrality (Figure 1f) reflecting the role of chaperone heat-shock proteins in multiple biological functions. Neuron-specific gamma-enolase (ENO2 or NSE) with a catalytic role in the synthesis of pyruvate and CALB2 (calretinin; a neuron-specific calcium-binding protein) follow HSPA12A in betweenness centrality (Figure 1f). The consistent downregulation of proteins involved in cholesterol metabolism is very interesting and such mechanisms in SCI are not well understood. One likely hypothesis is their involvement in myelin synthesis [17] and as such, their restoration might promote myelination after SCI. In contrast, downregulation of cholesterol synthesis has been associated with the highly effective regenerative capacity that is observed in peripheral nerves [18].

### 2.3. Identification of Druggable Proteins in SCI

To examine further the tight list of 40 upregulated and 48 downregulated molecules, we first looked for proteins that could be targeted using currently available, clinically-approved or experimental drugs/bioactive chemicals and second, we asked whether these druggable proteins have been cited in studies related to SCI. Druggable proteins were predicted using DGIdb v3 [19] and further validated with StitchDB v5 [20]. The relevance of druggable proteins to SCI was then examined using an automated PubMed text-mining tool that we developed for this study (source code deposited online: https://goo.gl/vRScJ3). Druggable proteins were searched in PubMed in conjunction to the terms “spinal cord injury” and “spinal injury” as well as with the words “trauma,” “contusion” or “transection” replacing “injury” in the query.

**Figure 2:**
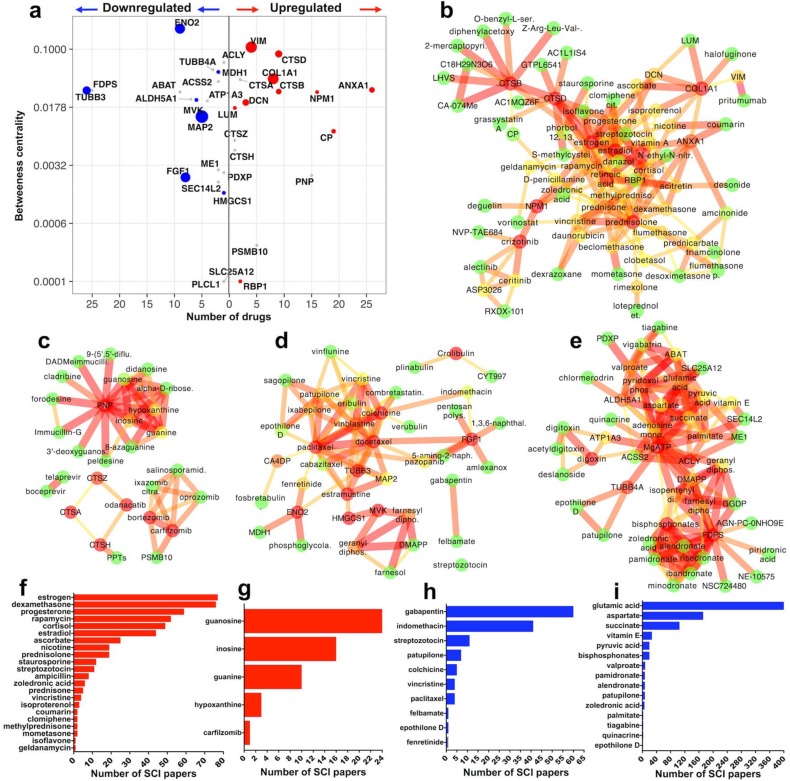
Mining druggable proteins using DGIdb and StitchDB: (**a**) DGIdb analysis followed by StitchDB identified 15 upregulated druggable proteins 10 of which returned at least one citation in SCI and 19 downregulated druggable proteins 7 of which with at least one citation in SCI. Proteins are summarised in 3 dimensions. Druggable proteins are plotted against the number of drugs predicted to act against them (*x* axis) versus their network betweenness centrality (*y* axis) to visualise druggability versus relative biological importance. The size of nodes (3rd dimension) indicates the number of PubMed articles citing these proteins in SCI. Grey nodes are proteins with no SCI citations in PubMed. (**b**) Protein-protein-drug interaction network of the 10 upregulated druggable proteins with at least one citation in SCI. The network was made using StitchDB and visualised in Cytoscape. Nodes and edges are colour-coded according to their betweenness centrality while the width and colour of edges indicates the strength of interaction between molecules (proteins and drugs) as predicted by StitchDB. (**c**) Protein-protein-drug interaction network of the 5 upregulated druggable proteins with no citations in SCI. (**d**) Protein-protein-drug interaction network of the 7 downregulated druggable proteins with at least one citation in SCI. (**e**) Protein-protein-drug interaction network of the 12 upregulated druggable proteins with no citations in SCI. (**f**–**i**) Predicted drugs from networks were text-mined in PubMed for potential reference to SCI and the number of articles is presented; (**f**) drugs interacting with proteins in network (**b**); (**g**) drugs interacting with proteins in network (**c**); (**h**) drugs interacting with proteins in network (**d**); (**i**) drugs interacting with proteins in network (**e**).

DGIdb and StitchDB identified 15 upregulated druggable proteins, 10 of which returned at least one citation in SCI and 19 downregulated druggable proteins, 7 with at least one citation in SCI. These are summarised in Figure 2a. Druggable proteins are plotted against the number of drugs predicted to act against them (*x* axis) versus their betweenness centrality (*y* axis), to visualise druggability versus relative biological importance Figure 2a. Next, StitchDB-validated druggable proteins and their protein-protein as well as protein-drug interactions are illustrated in 4 networks. Figure 2b: 10 upregulated proteins with SCI citations plus 60 associated drugs; Figure 2c: 5 upregulated proteins with no SCI references plus 22 associated drugs; Figure 2d: 7 downregulated proteins with SCI citations plus 33 associated drugs; Figure 2e: 12 downregulated druggable proteins with no SCI citations plus 34 linked drugs. The predicted drugs from networks in Figure 2b–e were also text-mined in PubMed for potential reference to SCI (as described above) and the number of retrieved articles is shown in Figure 2f–i. All identified drugs from each network (Figure 2b–e) are listed here (https://goo.gl/zwbuq1) together with the number of citations retrieved from text-mining using SCI terms (as explained above) and compared to terms “brain,” “spinal cord” and “central nervous system” for comparison. Links to the drug database PubChem is also provided for fast screening of essential drug information.

#### 2.3.1. Upregulated Druggable Proteins with SCI Citations (Figure 2b,f)

In the group of upregulated proteins with SCI citations (Figure 2b), the association of annexin A1 (ANXA1) with classic corticosteroids (cortisone, dexamathasone, prednisone, amcinonide and associated steroids and hormones, i.e. estradiol and progesterone) dominates the network (Figure 2b). The potent anti-inflammatory effect of corticosteroids in tissues is thought to be exerted, at least in part, by regulating the synthesis and function of ANXA1 [21]. Interestingly, while ANXA1 has received limited attention in SCI, corticosteroids have been extensively used to reduce acute SCI inflammation (Figure 2f) but their efficacy in alleviating long-term pathology is debatable and their use is controversial [22,23]. Steroid hormones estradiol and progesterone are also well studied (Figure 2f) and are considered neuroprotective in SCI [24].

Another notable cluster in Figure 2b includes the well-studied cathepsins B and D (CTSB, CTSD) and the numerous mostly experimental inhibitors that block their proteolytic activity. Both CTSB and CTSD are likely involved in the degradation of axonal components after SCI [25] but notably anti-cathepsin drugs have not been used in SCI thus far in either preclinical or clinical studies. NPM1 (nucleophosmin; Figure 2b) is associated with anti-neoplastic drugs crizotinib and deguelin (plus associated compounds). While inhibition of NPM1 was recently noted to block neuronal apoptosis after SCI in one study [26], neither deguelin nor crizotinib have been tested in SCI (Figure 2f).

Although vimentin (VIM; Figure 2b) has been cited extensively in SCI, mainly as a non-specific marker of proliferating (astrocytes, ependymal cells, fibroblasts) or invading (macrophages, endothelial cells, progenitors) mesenchymal cells in the injury site, it has not been tested as a putative drug target thus far. The cytoskeletal protein, which has very high betweenness centrality (Figure 2a), is recently gaining attention as a potential target in glioma and other cancers using antibodies against its ectodomain (pritumumab [27]). In SCI, anti-vimentin antibodies could be conceivably used for the removal of vimentin-positive cells but the selectivity of such an approach against a protein that is highly abundant in multiple cell types, some with potentially neuroprotective roles [28], is questionable.

The archetypal matrix and fibrosis protein collagen-1 (chain COL1A1; Figure 2b) is persistently upregulated after SCI, it has high betweenness centrality (Figure 2f) and is associated with the anti-fibrosis collagen expression blocker halofuginone [29], as well as with the beta-adrenergic receptor agonist isoproterenol (Figure 2b), known to increase its expression [30]. Notably, this is one of the very few cases of an agonist present in our analysis. The potential involvement of collagen-1 in matrix remodelling, fibrosis and scarring after SCI is a promising area for future investigation.

#### 2.3.2. Upregulated Druggable Proteins without SCI Citations (Figure 2c,g)

In this small network the extensive druggability of PNP (purine nucleoside phosphorylase), an enzyme that converts ribonucleosides into purine bases, is striking. PNP has been implicated as an inflammatory mediator in glia as well as peripheral T and B-cells and is a good target for anti-inflammatory therapies especially with regards to T-cell activity [31,32,33]. PNP has not been studied in SCI and might represent an interesting drug target given its persistent upregulation, its involvement in inflammatory mechanisms and the sizeable cohort of associated drugs (Figure 2c) that can block its function (i.e. immucillins and forodesine). Interestingly, purine derivatives that associate with PNP (guanosine, inosine, hypoxanthine) are used in experimental SCI as neuroprotective/neurotrophic agents but this is independent to their biological connection to PNP [34]. 

Unlike cathepsins B and D (CTSB, CTSD) discussed above, the function of upregulated CTSA, CTSH and CTSZ in SCI is unknown (Figure 2c). Similarly, neither specific cathepsin blockers (PPTs: odanacatib, toluenesulfonic acid) nor other protease inhibitors (telaprevir, boceprevir), have been tested in SCI. CTSA, CTSH and CTSZ are all persistently upregulated after SCI and CTSA has high betweenness centrality (Figure 2a). Thus, the availability of cathepsin blockers (Figure 2c) and the lack of knowledge about these cathepsins make them interesting candidates for further investigation. In addition to cathepsins, the role of the proteasome protease PSMB10 (Figure 2c) in the spinal cord is not studied and not much is known about the proteasome-ubiquitin system in SCI [35]. The proteasome inhibitor carfilzomib which interacts with PSMB10 (Figure 2c), was recently shown to exert an acute neuroprotective effect after T10 transection SCI in rats (Figure 2g) but the authors did not implicate PSMB10 to this effect [36].

#### 2.3.3. Downregulated Druggable Proteins with SCI Citations (Figure 2d,h)

This dense network contains few multi-druggable entities (Figure 2d,h). The classic neuronal tubulin beta-3 (TUBB3) is associated with many cytoskeleton-regulating drugs, mainly paclitaxel (taxol) and derivatives (docetaxel, cabazitaxel, epothilone D, patupilone/epothilone B) plus other putative microtubule stabilisers like estramustine, vinblastine and vincristine (Figure 2d). Although mainly used as anti-neoplastic agents, microtubule stabilisers (notably paclitaxel and patupilone/epothilone B) are currently at the forefront of experimental SCI therapies [5,6]. Another microtubule assembly protein, MAP2 (microtubule-associated protein 2) is consistently downregulated after SCI (Figure 1d) and is also predicted to interact with these drugs (Figure 2d). MAP2 is a well-studied neuronal marker. It has been previously shown to be downregulated after SCI and its loss is associated with destabilization and depolymerization of axonal microtubules [37].

The activity of the acidic fibroblast growth factor FGF1 can be blocked by the tyrosine kinase inhibitor pazopanib (FGF1 activates tyrosine kinase signalling) and by the anti-inflammatory amlexanox (Figure 2d) both with no citations in SCI. Nevertheless, the use of drugs that block FGF1 activity is counterintuitive, as it is persistently downregulated in our analysis plus it has been shown to be neurotrophic and neurorestorative after SCI in preclinical studies [38]. Like FGF1, neuron-specific enolase (ENO2/NSE; Figure 2d) has broad neuroprotective function in the central nervous system [39]. ENO2 is a protein with high betweenness centrality (Figure 2a) and potentially high biological importance. The anti-cancer drug fenretinide has been found to interact with enolase (Figure 2d) but the exact pharmacological action is unknown. Interestingly, fenretinide was recently shown to alleviate inflammation in murine contusion SCI but the authors did not connect this effect to ENO2 [40].

Key downregulated sterol synthesis enzymes MVK and HMGCS1 interact with the chemical farnesol (Figure 2d) but potential pharmacological effects are unclear. They also interact with substrates geranyl and farnesyl diphosphate, again with unknown pharmacological benefit. The role of MVK and HMGCS1 is unclear but cholesterol metabolism and the mevalonate cascade are recently gaining attention in the field [41].

#### 2.3.4. Downregulated Druggable Proteins without SCI Citations (Figure 2e,i)

In conjunction with MVK and HMGCS1 from (Figure 2d), farnesyl diphosphate/pyrophosphate synthase (FDPS) is a critical mevalonate synthesis enzyme with multiple established biphosphonates (i.e., alendronate, etidronate, etc.) as well as experimental (NE-10575, NSC724480) inhibitors (Figure 2e) and medium betweenness centrality (Figure 2a). Yet, the enzyme has not been studied in SCI and its function in the brain and spinal cord remains elusive [42]. In contrast, clinical and experimental inhibition of FDPS by biphosphonates is the gold-standard approach to block bone resorption by inhibiting the function of osteoclasts. As a result, biphosphonates are currently used to regulate inactivity-induced bone resorption in SCI patients [43] and this is independent to any potential function of FDPS in the spinal cord. ACSS2 and ACLY are important lipid synthesis mediators with high betweenness centrality but unknown role in SCI (Figure 2a). They are prominent in the drug network (Figure 2e), mainly due to their interaction with adenosine phosphate (and MgATP) and lipid metabolism intermediaries palmitate, succinate and pyruvate. Potential therapeutic effects are unknown. Notably, ACLY is involved in the synthesis of the neurotransmitter acetylcholine in the brain [44]. ACLY is also associated with ME1 (malic enzyme; Figure 2e) which generates NADPH for fatty acid synthesis. The role of ME1 in the spinal cord is also unknown but it was recently shown, together with other lipid synthesis molecules, to have a possible function in white matter development in infants [45].

ATP1A3 (Figure 2e), a sodium/potassium pump ATPase is another enzyme with unknown function in SCI and high betweenness centrality (Figure 2a). The enzyme has a distinct neuronal function as it is involved in the generation of electrical impulses and in the transport of neurotransmitters and calcium ions across the plasma membrane [46,47]. It is associated with the cardiac glycoside digoxin/digitoxin and derivatives, which have not been tested as SCI treatments and are rarely used in modern clinical practice to enhance cardiac function.

Two highly druggable, downregulated proteins with high betweenness centrality are aldehyde dehydrogenase 5A (ALDH5A1) and 4-aminobutyrate aminotransferase (ABAT) (Figure 2a,e) both involved in the catabolism of the inhibitory neurotransmitter GABA [48,49]. Interestingly, although the role of these GABA catabolic enzymes in SCI is unknown, the associated drug valproate (plus associated vigabatrin, chlormerodrin and tiagabine) are used (Figure 2i) to increase GABA and prevent the spasticity and involuntary muscle contractility that affects the majority of SCI patients [50]. Thus, it could be speculated that the persistent downregulation of ALDH5A1 and ABAT described here, might contribute to the molecular mechanisms of post-SCI spasticity which is nevertheless caused by very complex neuronal mechanisms following injury to upper or lower motor neurons. 

#### 2.3.5. Transcription Factor Regulation of Persistently Differentially Regulated Proteins

**Figure 3:**
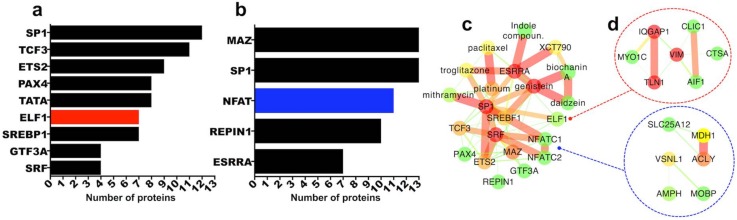
Transcription factor promoter binding site analysis for 40 and 48 persistently differentially regulated proteins: (**a**,**b**) MSigDB was used to identify transcription factors with likely promoter binding sites for the molecules that were consistently upregulated (**a**) or downregulated (**b**) in all 4 transcriptomics and proteomics datasets, at both 7 days and 8 weeks post-SCI. Graphs display the number of proteins (*x* axis) likely regulated by listed transcription factors. ELF1 is upregulated while NFATC1 is downregulated in both 7 days and 8 weeks transcriptomics datasets. (**c**) Protein-protein-drug interaction network made in StitchDB and Cytoscape depicting interacting transcription factors (from **a**,**b**) and associated drugs. Neither ELF1 nor NFATC1 have predicted drugs with either DGIdb or StitchDB. The upregulated (red) and downregulated proteins with likely promoter binding sites for ELF1 and NFATC1 are highlighted in (**d**).

One approach to regulate gene and protein expression in tissues is via interference with relevant transcription factor activity. Given that our combined intersection of transcriptomics and proteomics datasets isolated a filtered list of 40 significantly upregulated and 48 significantly downregulated proteins both at the mRNA and protein level and both at 7 days and 8 weeks post-SCI (Figure 1), we sought to examine likely transcription factor promoter-binding sites for the identified proteins. To do this, we used TRANSFAC-7-based computational prediction for transcription factor promoter binding sites (MSigDB; [51]). This analysis returned 9 transcription factors as potential regulators of the 40 upregulated proteins (Figure 3a) and 5 transcription factors potentially controlling the expression of the 48 downregulated proteins (Figure 3b).

SP1 followed by TCF3 (or TFE2) are predicted to bind to the highest number of upregulated gene promoters (Figure 3a) while MAZ and SP1 are predicted for the highest number of downregulated genes (Figure 3b). These transcription factors are conserved and abundant housekeepers and regulate a plethora of genes, hence their involvement is not surprising. Nevertheless, potential specific role of either MAZ or SP1 in SCI is currently unknown. 

When we examined the expression profile of these transcription factors in the transcriptomics and proteomics datasets we found that although none were differentially regulated at the protein level (proteomics datasets) ELF1 which was predicted to regulate 7 of the 40 persistently upregulated proteins (Figure 3a), was itself upregulated at the mRNA level both at 7 days and 8 weeks post-SCI (7 days SCI microarray: https://goo.gl/XqbbgN; 8 weeks SCI microarray: https://goo.gl/BXYEeT). On the other hand, NFAT transcription factor complex promoter binding sites were predicted for 11 of the 48 persistently downregulated proteins (Figure 3b) and NFATC1, one of the NFAT components, was downregulated at the mRNA level at both 7 days and 8 weeks post-SCI (7 days SCI microarray: https://goo.gl/XqbbgN; 8 weeks SCI microarray: https://goo.gl/BXYEeT). As for SP1 and MAZ (both upregulated at the mRNA level at 7 days but not 8 weeks), the potential function of either ELF1 or NFATC1 in SCI is unknown. Upregulated proteins with predicted regulation by ELF1 and downregulated proteins with predicted regulation by NFATC1 are depicted in Figure 3d. Predicted transcription factors from Figure 3a,b were also examined using DGIdb and StitchDB for potential association with drugs (Figure 3c). Neither ELF1 nor NFATC1 appear to be druggable. SP1 and ESRRA, are instead associated with anti-cancer compounds (Figure 3c) which have not been tested in SCI thus far. Interestingly, the only transcription factor found in the filtered list of the 40 upregulated molecules at the mRNA and protein level, 7 days and 8 weeks post-SCI, is STAT1 (signal transducer and activator of transcription 1), a key regulator of inflammatory mechanisms and interferon signalling in particular [52,53]. STAT1 is persistently upregulated after SCI and has a high betweenness centrality (Figure 1a–c). Although it has been involved in various inflammatory mechanisms and diseases, its function after SCI is not well-studied, albeit few recent studies have shown that blocking STAT1 activity has a positive effect post-SCI [54,55]. To the best of our knowledge, no approved drugs or inhibitors offer selective inhibition of STAT1. 

## 3. Discussion

In this manuscript combination of transcriptomics, proteomics and bioinformatics provides a comprehensive overview of proteins with persistent differential expression at the mRNA and protein level and from the subacute (7 days) to the chronic (8 weeks) phase of SCI lesion development. To ensure stringent filtering, we accepted only molecules that were significantly differentially regulated in all high-throughput datasets (transcriptomics and proteomics, 7 days and 8 weeks post-SCI). The intersection of transcriptomics and proteomics is useful given that while mRNA screening provides a snapshot of the dynamic gene expression changes following SCI, proteomics confirms functional expression of proteins from regulated transcripts and underscores proteotypic changes in the injured spinal cord. To the best of our knowledge, this is the first attempt to systematically analyse the molecular druggability of SCI in high-throughput.

The intersection of transcriptomics and proteomics at 2 different injury time-points and from 2 independent labs using comparable contusion and compression SCI models in female rats, ensures unbiased validation of the high-throughput data and offers confidence in the filtering of these molecular targets. Nevertheless, it is important to note that the transcriptomics and proteomics data were based on two different SCI models as well as rat strains. More specifically, the transcriptomics analysis by Chamankhah et al. was based on a T7 aneurysm clip (35 g) compression injury (moderate to severe) in female Wistar rats [11], while our proteomics analysis was based on automated 150 kilo-dyne (1.5 Newton) spinal contusion injuries (moderate), performed on female Sprague-Dawley rats (see Section 4 for more details and [7,9,10,56]). It is important to note that different injury models might cause variable tissue pathology and sensorimotor outcomes. Differences and similarities between contusive and compressive injuries have not been studied in detail but both models are considered comparable, as they rely on blunt trauma to the spinal cord and normally involve neither penetration of the meninges, nor sharp severing of axons (as occurs in hemisections and full transections). As a result, they better simulate the biomechanical damage observed in the majority of human injuries (reviewed and summarized in [57,58,59,60]). Few studies have directly compared contusions with compressions. Pinzon et al., showed that gross lesion pathological features did not appear to differ greatly between contusion and compression injuries [61]. More recently, Geremia and colleagues concluded that lesion volumes and gross tissue pathology were not significantly different between contusion (severe T12, 200 kilo-dyne) and clip-compression (moderate to severe T12, 35 g aneurysm clip), albeit clearly increased hematoma formation and spreading in the severe contusion group [62]. Differences in contusion/compression forces applied in different studies are clearly important in terms of defining injury severity. Comparison between strains has revealed differences in a number of studies not only between strains but even in same strains obtained from different vendors [63]. 20 years ago Popovich and colleagues focused on differences in inflammation after SCI. They found that while the basic inflammatory reaction after SCI follows similar patterns in Sprague-Dawley and Lewis rats, differences were observed in the magnitude and duration of macrophage activation and T-cell infiltration in lesions. The authors attributed these differences to strain-specific variations in corticosteroid regulation of inflammation [64]. Accordingly, using a neuronal-specific gene array, Schmitt et al., showed that the expression of several genes varies between Sprague-Dawley, Long Evans and Lewis rats after contusive SCI [65] and in a study focusing on post-SCI sensorimotor function, Mills et al., showed that strain selection significantly affects functional recovery in Sprague-Dawley and Wistar rats [57]. Thus, different strains might exhibit pathological or sensorimotor differences. Nevertheless, our focus on proteins that are differentially regulated in both models, both time-points and both at the mRNA and protein level highlights the likely importance of these differentially regulated signatures to SCI.

Our high-throughput intersection returned 40 persistently upregulated and 48 persistently downregulated proteins. To further narrow down the list of target molecules, we applied two-tier computational protein-protein-drug interaction screening combined with literature text-mining. This analysis isolated previously unappreciated druggable proteins and pharmacological substances that could be examined in future experimental and preclinical SCI studies. Notably, excluding a few proteins that have been studied extensively in SCI (i.e., vimentin, IBA1, decorin, ceruloplasmin, CTSB, CTSD, tubulin beta-3, ENO2/NSE, FGF1, MAP2 and others) many proteins and drugs identified in our comparative analysis have received little to no experimental attention and some might be excellent targets for future investigations.

While it would be very difficult within the limits of this manuscript to cover in detail the function of each protein and their potential role in SCI, we have made an effort to describe the bioactivity of multiple druggable proteins and put it in the context of SCI where applicable (see Figure 2 and associated Results). Given that the majority of drugs are antagonists, inhibitors, or function-blockers, upregulated molecules are excellent primary candidates for drug targeting. In contrast, downregulated entities might be modulated with function agonists or over-expression with more complex molecular tools such as viruses or CrispR/CAS9. 

**Figure 4:**
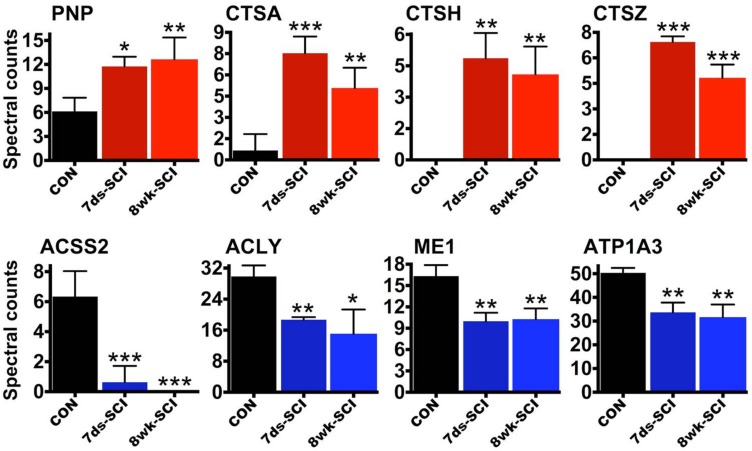
Highlighted differentially regulated proteins after SCI. Graphs depict the differential expression of upregulated purine nucleoside phosphorylase (PNP), cathepsins A, H and Z (CTSA, CTSH, CTSZ) and downregulated ACSS2 (acetyl-CoA synthetase), ATP citrate lyase (ACLY), malic enzyme (ME1) and sodium-potassium ATPase (ATP1A3) proteins using spectral counting values from shotgun liquid chromatography-tandem mass spectrometry (LC-MS/MS). These proteins are amongst the molecules that were consistently upregulated or downregulated at the mRNA and protein level and at 7 days and 8 weeks post-SCI. *n* = 3 per group; mean +SD; ANOVA and Fisher post-hoc test (independent comparison); stars indicate significance versus CON (control intact T10 spinal cord segments); * *p* ≤ 0.05, ** *p* ≤ 0.01, *** *p* ≤ 0.001. CTSA and CTSZ spectral counts are also significantly different from 7 days to 8 weeks.

Based on biological function, druggability and crucially, lack of knowledge with regards to SCI, we could highlight few interesting proteins that are consistently upregulated or downregulated at the mRNA and protein level, at 7 days and 8 weeks individually depicted in Figure 4. From the upregulated cohort, PNP (purine nucleoside phosphorylase) is a protein with well-documented involvement in T-cell activity and function and PNP inhibitors exert potent immunomodulatory effects against T-cells, especially in diseases with dominant involvement of adaptive immunity [32,66,67]. The role of PNP in B-cell function is also under investigation. Notably, not only the potential function of PNP in SCI is unknown but in addition, the role of T-cells is still unclear, although T-cells infiltrate spinal lesions [64]. Thus, given the importance of inflammation in primary and secondary SCI pathology and the importance of T-cells in immune processes, the highly druggable PNP (Figure 2c) is a very interesting candidate.

Similarly, the role of upregulated lysosomal cathepsin A (CTSA; Figure 4) in SCI is unknown. This serine protease and carboxypeptidase is involved in the activation of sialidase and its deficiency causes the lysosomal storage disease galactosialidosis [68]. It also seems to play an important role in autophagy, which is involved in the regulation of various inflammatory mechanisms including SCI [69]. Recent work in mice showed that CTSA deficiency is associated with a severe neurological phenotype [70]. Similarly, the role of the other consistently upregulated cathepsins CTSH and CTSZ (Figure 4) in SCI is unknown.

From the persistently downregulated proteins, lipid and sterol synthesis mediators including ACSS2, ACLY and ME1 (Figure 4) are attractive candidates for further research. They might be involved in lipid and energy homeostasis or in myelin synthesis post-injury. The distinctly neuronal ATP1A3 is another persistently downregulated protein (Figure 4) with very interesting properties given its involvement in neurotransmission [47]. The protein has been studied extensively for its function in the central nervous system but its role in SCI is unknown. Mutations that impair ATP1A3 activity cause rapid-onset dystonia in humans and permanent neurological dysfunction [71]. The enzyme is also downregulated after neonatal cortical injury in rats [46].

In summary, by using a combination of transcriptomics, proteomics and bioinformatics we isolated multiple proteins with drug-targeting potential and unknown function in SCI. While it might be difficult to speculate on the best possible drug target or identify a panacea for SCI, our systematic analysis might stimulate further mechanistic or therapeutic studies in the future. 

## 4. Materials and Methods

### 4.1. Rat Transcriptomics—Microarray Analysis of Rat SCI

The rat microarray datasets analysed and integrated in this manuscript were recently performed and published by Chamankhah and colleagues. Information about the ethical use of animals in this study and in-depth information about the clinically-relevant compressive SCI model utilised in their study has been published [11]. Briefly, the authors used a clip compression SCI in female Wistar rats using a 35 g aneurysm clip for 60 s producing a moderate to severe T7 SCI. The compression model produces comparable blunt-force gross pathology and lesion characteristics to the contusion model that we used on the thoracic spinal cord (see below). Microarray data is available online via the gene expression omnibus (GEO-NCBI). Microarray data from GEO is qualitatively and statistically curated and MIAME-compliant. Rat T7 spinal clip compression injury microarray *n* = 4 intact versus injured T7 spinal cord samples at 7 days (*n* = 4) and 8 weeks (*n* = 4) post-injury. Microarray experimental information is available here (GEO-NCBI: https://www.ncbi.nlm.nih.gov/geo/query/acc.cgi?acc=GSE45006). Additionally, microarray expression data was downloaded and compiled to excel files (.xlsx) to enable uncomplicated examination of differential mRNA expression between control and injured spinal cord specimens at 7 days and 8 weeks post-SCI and to allow investigation of individual genes. Due to their excessive size, these excel files have been publicly deposited and are free to download from the Mendeley Data public repository. Control versus 7 days post-SCI microarray comparison can be found here: (7 days SCI microarray: https://goo.gl/XqbbgN; 8 weeks SCI microarray: https://goo.gl/BXYEeT). Expression and statistical analysis was performed by GEO-NCBI using standard *t*-test *p*-values and multiple *p*-value error adjustments. We only accepted transcripts that were significant with an adjusted *p* ≤ 0.05 and 2-fold change at both 7 days and 8 weeks. 

### 4.2. Spinal Cord Injury Model in Rats for Proteomics Analysis

We have previously characterised in detail spinal cord contusion in adult rats [7,9,10,56]. Briefly, adult female Sprague-Dawley rats (~200 g) were anesthetised using breathable isofluorane. 5 mg/kg Baytril (antibiotic) and 5 mg/kg Carprofen (NSAID pain and inflammation control) were given subcutaneously at the time of surgery and the morning after surgery. Spinal laminectomies were performed at vertebral level T10, the vertebral column was stabilised using Adson forceps and the rat-specific impactor probe was positioned 2 mm above the spinal cord. An impact force of 150 kilo-dyne (mean 152.3 kilo-dyne; standard deviation 3.2; *n* = 6 contused animals) was delivered to the exposed spinal cord through the intact dura with an Infinite Horizon impactor (Precision Systems Instrumentation) which generates a moderate severity contusion injury according to our Home Office-approved animal licence. This severity mimics more than 50% of human injuries that are “incomplete” (i.e., where some white matter tissue is spared) containing uninjured axons and the model is generally considered to be relatively clinically-relevant. After injury, all 6 animals used in the study had full bilateral hind limb paralysis and started exhibiting limited spontaneous recovery typically after the first week. After spinal contusion, the overlying muscle and skin were sutured, anaesthesia was reversed using oxygen and animals recovered in cages placed on heated blankets. Saline and Baytril (antibiotic) were given subcutaneously daily for 7 days, after injury. Bladders were manually expressed twice daily until reflexive emptying returned (typically 6–9 days after injury). The study has received approval by the institutional Animal Care and Use Committee (King’s College London; PPL 70/8032, 14/11/2016) and all surgical procedures were performed in accordance with the United Kingdom Animals (Surgical Procedures) Act 1996.

### 4.3. Shotgun LC-MS/MS Proteomics—Proteomics Analysis of Rat SCI

We have recently described in detail and published high-resolution shotgun LC-MS/MS proteomics analysis of rat SCI [10]. Prior to proteomics spinal cord tissue from intact (*n* = 3) or injured T10 spinal cord segments 7 days (*n* = 3) and 8 weeks (*n* = 3) post-injury were extracted using a sequential protein extraction protocol previously developed by us [10,12,13]. This approach improves separation of easily soluble cellular proteins (0.08% SDS) followed by isolation of insoluble and cross-linked, extracellular, matrix and matrix-associated extracellular proteins from tissue specimens (4 M guanidine). This method has been published multiple times and is widely used for different tissues including the spinal cord. Following protein extraction shotgun LC-MS/MS was performed as described in depth [10]. Briefly, protein samples were digested by trypsin, tryptic peptides were separated in 2–35%, 120 min acetonitrile gradient and analysed on Q Exactive orbitrap mass spectrometer. Protein identifications were performed using Mascot Version 2.4.1 (Matrix Science). Scaffold [72] (version 4.2.1) was used to validate MS/MS based peptide and protein identifications. Peptide identifications were accepted only if they could be established at greater than 95.0% probability by the Peptide Prophet algorithm with Scaffold delta-mass correction. Protein identifications were accepted only if they could be established at greater than 95.0% probability and contained at least 2 unique identified peptides. Scaffold was also used to calculate normalised spectral counts for quantitation. All accepted protein spectra (95% probability plus 2 unique peptides minimum) were included in the analysis and no outliers were removed. Although there are multiple different approaches to achieve relative protein quantitation using LC-MS/MS, spectral counting is very simple and less prone to technical errors in comparison to protein-labelling approaches especially when combined with orthogonal validation of findings [10,14]. In this manuscript, extra confidence is obtained from the fact that we focus only on proteins that are common across 2 independent transcriptomics and proteomics datasets and across 2 different time-points. Only proteins with *t*-test *p* ≤ 0.05 at both 7 days and 8 weeks and concomitant significant differential regulation (*p* ≤ 0.05) at the transcript level (from microarrays described above) at both 7 days and 8 weeks were accepted. All proteomics identifications and spectral counting quantitation has been deposited online at Mendeley Data: 7 days proteomics: https://goo.gl/k93LwN; 8 weeks proteomics: https://goo.gl/qYoTJz.

### 4.4. Computational and Bioinformatics Analysis of High-Throughput Data

Hierarchical clustering and heat-maps were created in the MeV TM4 platform [73]. Significantly different (*p* ≤ 0.05; *t*-test) spectral counts were z-normalised (−3; low to +3; high) to obtain a more linear colour representation of the data. Pairwise similarity in spectral counts between different proteins (rows) was computed using Pearson correlation coefficient. Protein-protein interaction networks were created using StringDB v10 (https://string-db.org) [74] of known and predicted protein-protein interactions and inferring protein associations from multiple databases as well as text-mining. For protein-protein interaction networks, a low threshold of association (0.15) was used to capture the largest possible interaction probability. Network parameters were visualised in CytoScape v2.8 [75], which was also used to calculate the betweenness centrality of interacting proteins within networks. Drug candidate analysis was performed using DGIdb (http://www.dgidb.org) [19]. This software is searching multiple different drug databases and uses text-mining to identify drugs matching input proteins. Drug-protein interactions were then validated and filtered using StitchDB v5 (http://stitch.embl.de) [20]. This step resulted in significant filtering of the drug-protein interaction data. StitchDB also creates organic drug-protein and protein-protein interaction networks, the latter using the StringDB platform and generates interaction scores. For protein-drug interactions, a medium threshold of association (0.40) was used to ensure more stringent filtering of drugs and chemicals. As above, network parameters were visualised in CytoScape v2.8, which was also used to calculate the betweenness centrality of interacting proteins and drugs within networks. Transcription factor analysis was performed using MSigDB (http://software.broadinstitute.org/gsea/msigdb) [51], transcription factor targets sub-collection. Mammalian transcriptional regulatory motifs were extracted from v7.4 TRANSFAC database. Each gene set consists of all human genes whose promoters contains at least one conserved instance of the TRANSFAC motif, where a promoter is defined as the non-coding sequence contained within 4-kilobases from the transcription start site.

### 4.5. Gene and Drug Java Text-Mining Tool

The custom-made text-mining tool was constructed using Java in IntelliJ IDEA community edition. The code used has been publicly deposited in Mendeley Data for Journals and can be found here together with running instructions to operate the tool in IntelliJ for free (https://goo.gl/vRScJ3). Firstly, the nomenclature for the text-mined genes is retrieved using the HGNC REST web-service API (https://www.genenames.org/help/rest-web-service-help). The gene symbol, gene name and synonym identifiers were used in the literature queries. The gene nomenclature is enriched by a set of algorithms that are designed to permute names and omit obsolete name parts. The literature queries are performed using the Europe PubMed Central (EPMC) REST web-service API after nomenclature retrieval. The EPMC database queries are structured into two blocks; the first block contains all gene names, abbreviations, synonyms and accepted protein names from HGNC, whereas the second block contains the keywords of interest including: “spinal cord injury” and “spinal injury” as well as with the words “trauma,” “contusion” or “transection” replacing “injury” in the query. The search clause is structured so that at least one search term belonging to each of the search blocks must be present in the abstract or title of the retrieved articles. All search terms were enclosed in quotations to ensure that the terms are searched as is and that individual words within the term are not matched mistakenly. The retrieved articles were manually verified to satisfy search requirements. Drug text-mining was performed similarly. The drugs for the differentially regulated genes were derived manually with the use of DGIdb and StitchDB. Next, EPMC was queried in the same way as for the genes, where the first block of the query contained the primary drug names instead of gene names. This article set contained no false positives. Please note that different text-mining search engines such as Google Scholar might retrieve more or different studies regarding the usage of certain drugs in SCI but in our experience PubMed is more reliable with regards to peer-reviewed research-based studies and in our experience, retrieves fewer false-positives. A deeper text-mining was then performed using all drug synonyms, chemical names and commercial names to ensure a full, in-depth retrieval of literature. Drug synonyms were retrieved using the PubChem REST web-service APIs. The risk for the retrieval of false positive articles was high in this search due to the extensive variety of drug synonyms stored in the PubChem database. To avoid the retrieval of false positives, the results of the deep text mining were overlaid with the “drug name only” results and differences in article numbers between the two datasets were inspected manually and false positives eliminated. URL addresses for all online database queries (PubChem, HGNC, EPMC, UniProt) are output in the console at runtime. The user can verify database query parameters by navigating to these URLs manually. Please not that although every effort was made to minimise retrieval of false-positive associations via text-mining, users must ensure a stringent manual search of target molecules.

## Abbreviations

SCI	Spinal cord injury
LC-MS/MS	Liquid-chromatography and tandem mass-spectrometry
